# The Application of Nanobody in CAR-T Therapy

**DOI:** 10.3390/biom11020238

**Published:** 2021-02-08

**Authors:** Chaolemeng Bao, Quanli Gao, Lin-Lin Li, Lu Han, Bingxiang Zhang, Yijin Ding, Zongpei Song, Ruining Zhang, Jishuai Zhang, Xian-Hui Wu

**Affiliations:** 1 Shenzhen Pregene Biopharma Company Ltd., Shenzhen 518118, China; baoclm@pregene.com (C.B.); zhangbx@pregene.com (B.Z.); dingyj@pregene.com (Y.D.); songzp@pregene.com (Z.S.); zhangrn@pregene.com (R.Z.); zhangjs@pregene.com (J.Z.); 2 Department of Immunology, Affiliated Cancer Hospital of Zhengzhou University and Henan Cancer Hospital, Zhengzhou 450008, China; zlyygql0855@zzu.edu.cn (Q.G.); LIN15838169920@163.com (L.-L.L.); zlyyhl2407@zzu.edu.cn (L.H.); 3 Department of Immunology, School of Basic Medical Sciences, Xinxiang Medical University, Xinxiang 453000, China

**Keywords:** nanobody, V_H_H, CAR-T, BCMA

## Abstract

Chimeric antigen receptor (CAR) T therapy represents a form of immune cellular therapy with clinical efficacy and a specific target. A typical chimeric antigen receptor (CAR) construct consists of an antigen binding domain, a transmembrane domain, and a cytoplasmic domain. Nanobodies have been widely applied as the antigen binding domain of CAR-T due to their small size, optimal stability, high affinity, and manufacturing feasibility. The nanobody-based CAR structure has shown a proven function in more than ten different tumor-specific targets. After being transduced in Jurkat cells, natural killer cells, or primary T cells, the resulting nanobody-based CAR-T or CAR-NK cells demonstrate anti-tumor effects both in vitro and in vivo. Interestingly, anti-BCMA CAR-T modulated by a single nanobody or bi-valent nanobody displays comparable clinical effects with that of single-chain variable fragment (scFv)-modulated CAR-T. The application of nanobodies in CAR-T therapy has been well demonstrated from bench to bedside and displays great potential in forming advanced CAR-T for more challenging tasks.

## 1. Introduction

Cell therapies encompass a vast number of agents in immuno-oncology development [1]. In various types of cell therapy, the chimeric antigen receptor (CAR) T cell is the most dominant and active anti-tumor agent in the treatment of cancers, especially in hematological malignancies [2], while limited activity has been shown in solid tumors and several challenges need to be overcome regarding its application [3]. The CAR structure is a genetically engineered molecule that direct T cells to specifically attack tumor cells through an antibody–antigen interaction rather than in a major histocompatibility complex (MHC)-dependent manner. Three CAR-T cell-based therapies, tisagenlecleucel (Kymriah), axicabtagene ciloleucel (Yescarta), and brexucabtagene autoleucel (Tecartus), have so far been approved by major regulatory agencies on B-cell precursor acute lymphoblastic leukemia (B-ALL)—that is refractory or in second or later relapse [4], r/r large B-cell lymphoma [5], and r/r Mantle-Cell Lymphoma [6]. All three CAR-T therapies target overexpressed CD19 membrane proteins.

## 2. Brief History of CAR T Cell Development

**Figure 1:**
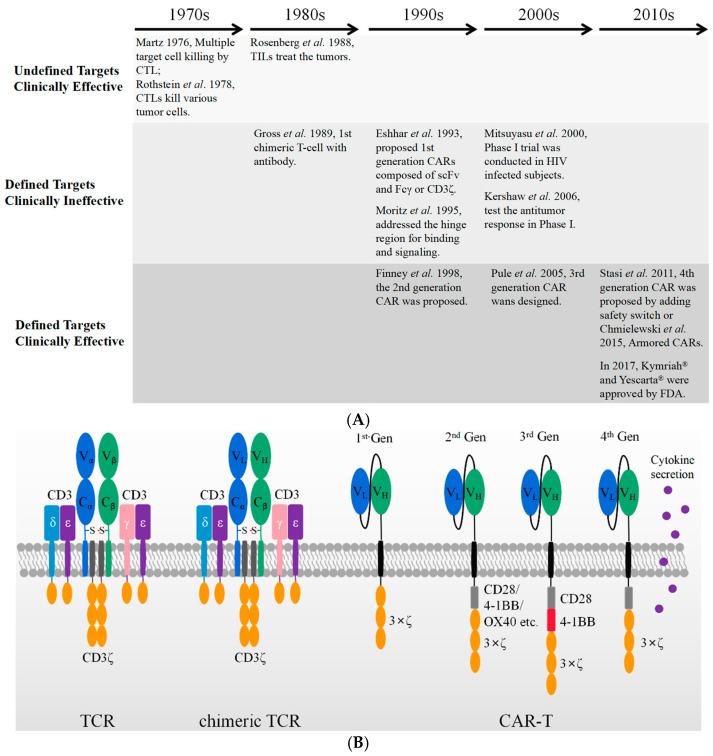
A brief history of cellular immunotherapy development from bench to bedside. (**A**) The CAR-T therapies evolved from undefined targets/clinically effective, defined targets/clinically ineffective, to defined targets/clinically effective. (**B**) The schematic figures of TCR machinery, chimeric TCR machinery, and different generations of CAR-T with the use of scFv.

The antitumor potential of T cells was realized about fifty years ago. It involved three stages, as shown in Figure 1A. It started from undefined targets/clinically effective cytotoxic T lymphocytes (CTLs) and tumor infiltrating lymphocytes (TILs) therapies, evolved into target-specific but clinically ineffective T-body and 1st generation CAR-T, and eventually became target-specific and clinically effective CAR-T therapies. In the 1970s, cytotoxic T lymphocytes (CTLs) were initially discovered to kill a variety of tumor cells, including P815 mastocytoma cells and EL4 lymphoma cells [7,8]. These reports reveal the potential application of T lymphocytes in cancer therapy. Tumor infiltrating lymphocytes (TILs) harvested from human dissected tumor tissues formed a straightforward application. These TILs were cultured ex vivo for four to eight weeks [9] and then re-administered intravenously along with a dose of IL-2 [10]. The clinical treatment exhibited encouraging effects on the regression of metastatic melanoma in 60% of patients enrolled [11]. The autologous CTLs and TILs are effective as therapeutics upon multiple undefined targets. Variable neo-antigens have been developed to simultaneously stimulate T cells for the treatment of solid tumors, and these are believed to achieve better clinical outcomes by overcoming the tumor heterogeneity [12]. However, their routine application still encounters challenges in manufacturing and prohibitive cost [13].

Target-specific T cells with chimeric receptors were firstly proposed by Zelig Eshhar and Gideon Gross in 1989 [14]. The V_α_ and V_β_ domain of αβ- T-cell receptor (TCR) were substituted by V_H_ and V_L_ of an anti-2,4,6-trinitrophenol (TNP) antibody, respectively. The resultant construct generated an artificial and chimeric TCR in the form of either V_H_-C_α_/V_L_-C_β_ or V_L_-C_α_/V_H_-C_β_, as shown in Figure 1B. The engineered receptor was a chimeric TCR, which is capable of binding TNP-coated target cells and activating cell lysis through the TCR machinery in an MHC-independent manner. As shown in Figure 1B, the first generation of CAR was designed by adjoining the scFv of the TNP antibody, a transmembrane domain, and the cytoplasmic immunoreceptor tyrosine-based activation motif (ITAM) domain of the CD3ξ or immunoglobulin Fc receptor γ-chain. T cells expressing such a genetically engineered first-generation CAR construct were also named T-body [15]. Although they could specifically kill the targeted tumor cells in vitro and in vivo in mouse models, these first-generation CAR-T cells showed a low cytotoxicity [16,17] and lacked the ability of long persistence in vivo [18]. Thus, they were not yet clinically effective.

The inability of long-lasting circulation in vivo was rapidly realized to be caused by the lack of the essential costimulatory receptor, as tumor cells rarely express a costimulatory receptor ligand such as B7 [19]. Therefore, in the second-generation CAR a cytoplasmic signaling domain from a costimulatory receptor, such as CD28 [20], 4-1BB [21]. or OX40 [22], was deliberately inserted between the transmembrane sequence and the ITAM domain of CD3ξ. These constructs display an optimized T cell activation, increased antigen-dependent proliferation in vitro [23], enhanced in vivo persistence, and more effective anti-tumor activity [24,25]. The second-generation CARs with a CD28 or 4-1BB costimulatory domain are currently well-established and the three FDA-approved anti-CD19 CAR-T cells are based on such constructs [4,5,6].

To further increase the T cell activation, a third-generation CAR was developed by incorporating an additional co-stimulatory domain into the second-generation CAR. Their typical cytoplastic domains are composed of CD28/4-1BB/CD3ξ [25] or CD28/OX40/CD3ξ [26]. Two clinical trials using third-generation CARs (CD28/4-1BB/CD3ξ) have been published on leukemia [27] and Non-Hodgkin lymphoma (NHL) [28], respectively. In both trials, the CAR-T cells transduced with third-generation CARs reveal a superior expansion and longer persistence, particularly in patients with a low disease burden and low levels of normal B cells [28]. Nonetheless, the number of patients treated by third-generation CAR-T is not large enough to lead to a conclusion.

Although clinical trials show the persistent efficacy of CAR-T in treating hematologic malignancies, the treatment of solid tumors has encountered major challenges. Solid tumors impede the efficient penetration of T cells and demonstrate a hostile micro-environment, which limits the effective cytotoxicity from CAR-T to the target tumor cells [29,30]. Therefore, the efficacy of CAR-T in solid tumors seems to depend on its enhanced cytotoxicity, longer persistence, and modulation of the tumor microenvironment by secreting cytokines simultaneously. The fourth-generation CARs were designed by adding new functions beyond T cell activation signals, including the secretion of a variety of cytokines and extra antibodies [31]. In particular, co-expressed cytokines and antibodies are believed to modulate the immune microenvironment within the solid tumor tissues. Such T-cells redirected for antigen-unrestricted cytokine-initiated killing (TRUCKs) combine the direct antitumor attack of CAR-T cells with the immune-modulating capacities of the delivered cytokines, including IL-7, IL-12, IL-15, IL-18, IL-23, and combinations thereof [31]. In addition, the CAR-T cell-directed release of these proteins ensures their local release to the targeted tumors and avoid the risky adverse events that can potentially be caused by systemic administration. 

For instance, the systemic administration of IL12 is highly toxic, thus limiting its clinic application at therapeutically effective doses [32]. IL-12 is majorly responsible for the induction and enhancement of cell-mediated immunity. Its capabilities include activating the cytotoxicity of natural killer (NK) and T cells by inducing INF-γ release, inhibiting and reprogramming immunosuppressive cells, and upgrading the expression of MHC [33,34]. The local release of IL12 with the use of CAR-T cells is suggested to strongly modify the tumor microenvironment by avoiding the toxicity of systematic administration [35,36].

CD19 CAR-T cells maintain a less differentiated phenotype and improve metabolic fitness, as has been seen ex vivo in the presence of IL15, which results in superior in vivo antitumor activity [37]. In this case, IL15 is suggested to be a promising application in armored CAR-T cells.

IL18 is a proinflammatory cytokine that belongs to the IL-1 cytokine family. It plays a role in inducing INF-γ release with the use of IL12 or IL-15. This property makes IL-18 a promising candidate for enhancing the anti-tumor efficacy of genetically modified T cells. IL18-secreting CAR-T cells exhibit their enhanced expansion, persistence in vivo, and enhanced antitumor activity in xenograft models [38,39].

Besides CAR-T therapy, TCR-T therapy also modifies the patient’s own T lymphocytes ex vivo but also applies T cell receptors (TCRs) [40]. TCR is a heterodimeric membrane protein composed of α- and β-chains. Antigen recognition by αβ-TCR depends on HLA, which presents intracellular peptide fragments. Thus, TCR-T cell therapy has a wider range of targets. However, TCR-T cell therapy is HLA-restricted not only in its presentation but also in its activate T cell function [13].

## 3. The Antigen Binding Domain of CARs

Thus far, the market has authorized three CAR-T cell products that all target CD19 and rely on scFv derived from the same murine monoclonal antibody, FMC63. The variable heavy-chain V_H_ and light-chain V_L_ are linked by a (GGGGS)_3_ sequence, and the resulting scFv fulfills the role of antigen binding. The scFv is a widely accepted format to develop both CAR-T therapies and bispecific antibodies due to its compact size, high affinity, and specificity maintenance in antigen recognition [41]. However, the form of scFv may partially compromise its antigen binding capacity and stability. Compared with Fab, V_H_ and V_L_ in scFv lack stabilization elements/structure through the constant domains of CH_1_ and C_L_. Meanwhile, the hydrophobic patches separating from the constant domains are exposed and need further engineering [42]. 

**Figure 2:**
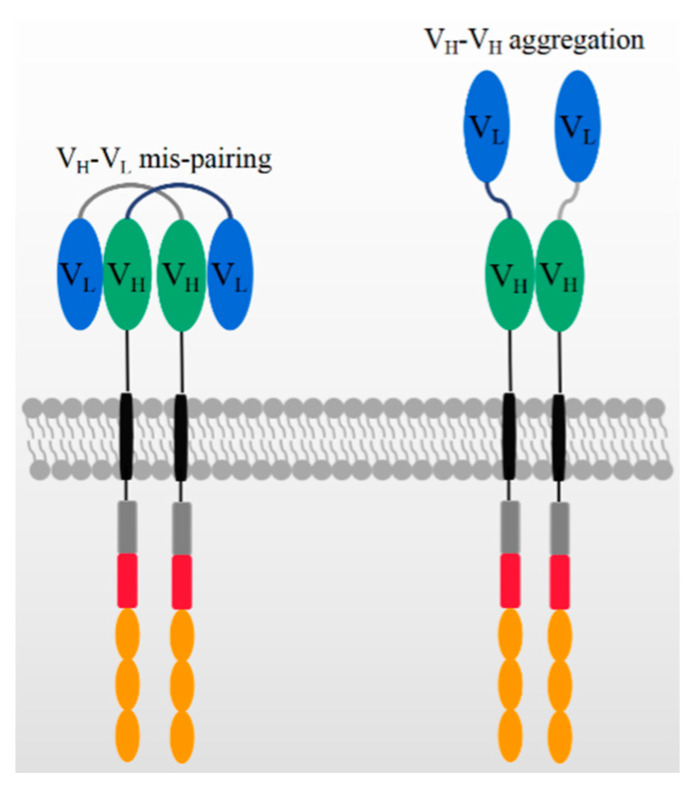
The mechanism of potential V_H_-V_L_ mispairing and V_H_-V_H_ aggregation on high CAR expression levels.

In addition to the antigen binding capacity and stability, the structure of scFv may also give rise to other potential risks and challenges in its application. A compatible linker sequence is required to connect V_H_ and V_L_ [43], and the linker sequence as well as the murine framework are the origin of immunogenicity risks that may lead to the generation of anti-drug antibodies (ADA) in vivo. The ADA effect could neutralize CAR-T cells’ functions and cause serious side effects, CAR-T cell loss, and even the failure of CAR-T therapy [44,45]. In ex vivo expansion, T cell exhaustion typically occurs due to CAR aggregation in an antigen-independent manner [44,46,47], which is probably triggered by the variable domains of scFv, as shown in Figure 2 [46]. This is consistent with previous reports that scFvs have a high propensity for self-aggregation because the hydrophobic patches are exposed on variable domains after deleting constant domains [42,47]. 

On the other hand, the structure of scFv may limit its potential for constructing more complicated CAR structures. Generally, bi-specific CARs can be constructed from two tandem antigen binding domains, referred to as TanCAR, which can recognize two different antigens or one/two epitopes on one antigen. In constructing TanCARs, the potential cross-pairing of V_H_ and V_L_ among two independent scFv molecules results in affinity loss [48]. In addition, multiple scFvs may influence the manufacturing because the size of the inserted gene compromises the viral packaging efficiency [49,50,51]. As shown in Figure 2, the over expression of CAR genes can result in the dynamic swapping of V_H_-V_L_ domains between different CAR units and aggregation on the cell surface. scFv aggregation or misfolding could be caused by low folding stabilities of the V_H_ or V_L_ domain or the exposure of hydrophobic residues at the V_H_–V_L_ interface [52,53]. The aggregation of CARs may induce excessive cytotoxic signaling independent of tumor antigens and eventually cause the early exhaustion of T cells [54]. 

As an alternative to scFv, using nanobodies in CAR-T constructs may attenuate the above-mentioned disadvantages. The nanobody, also called V_H_H antibody, is derived from the variable domain of heavy chain-only antibodies (HcAbs). The natural existence of functional heavy chain-only antibodies was found firstly in dromedaries by Hamers-Castermans et al. in 1993 [55] and has been widely found in Camelidae [56] and sharks [57] since then. The antigen recognition site of the HcAbs is the variable domain of the heavy chain of the HcAbs, referred to as V_H_H. Although a nanobody binds to the antigen in the complete absence of a V_L_ domain and constant domain, the binding capacity, specificity, solubility, and stability are comparable with those of traditional antibodies [56]. In addition, nanobodies are often composed of a long CDR3 sequence [58], which accepts an adjusted flexible and extended conformation. It is capable of reaching certain epitopes inaccessible to conventional antibodies [59], such as the active sites of enzymes and GPCRs [60]. 

In addition to its naturally high binding capacity, V_H_H also has a more favorable structure in terms of its in vivo immunogenicity, solubility, and stability. The high sequence similarity of V_H_H to the human V_H_ gene family III makes it more compatible for human use and less immunogenic in vivo [56]. Typically, nanobodies only require minor sequence amendments for a humanization process [61]. Furthermore, without light chains and CH_1_ domains, the V_H_H domain is more soluble and stable as compared with those of V_H_ and V_L_ in the traditional antibodies. Compared with scFv, V_H_H antibodies avoid the potential disrupted interaction between variable domains (V_H_, V_L_) and constant domains (CH_1_, C_L_) and the exposure of hydrophobic patches. Such disrupted interaction and hydrophobic residues may severely affect the solubility and stability [42]. Due to these properties, the nanobody holds a unique potential in developing various forms of CAR-T [62], and many studies have explored nanobody-based CAR constructs over the years [63]. 

## 4. The Applications of Nanobodies in CAR-T Therapies

As listed in Table 1, many efforts have already been made to develop CAR-T therapies using nanobodies because of their widely recognized advantages. The first published CAR-modified T cell with a nanobody utilized the anti-MUC1 V_H_H as the target binding domain [64]. The retrieved nanobody was joined with the human IgG3 hinge and IgG3-Fc as a spacer, while CD28 and CD3ζ were introduced as signaling domains. After the CAR has been transduced in Jurkat cells, an increased proliferation was observed upon co-culturing with MUC1^+^ MCF7 tumor cells. Moreover, the transduced Jurkat cells showed activity in cell lysis and IL2 secretion. Even though different hinges, such as IgG3-Fc-hinge and IgG3-Fc-hinge-hinge, were utilized in constructing CAR, the corresponding engineered Jurkat T cells maintain a similar IL2 secretion and proliferation upon the stimulation. However, the CAR with the use of an FcγRII hinge revealed a significant reduction in CAR expression and IL2 secretion.

The PhiC31 integrase system was further employed to optimize the CAR transduction and expression efficiency, which is encoded by a phage of streptomyces soil bacteria [79]. It is able to integrate introduced plasmid DNA into preferred locations in unmodified mammalian genomes, resulting in the robust, long-term expression of the integrated transgene [80]. By flow cytometry, the expression of anti-MUC1 CAR has been detected on the surface of Jurkat cells at day 1 and 30 after transfection. At day 1, there is not much difference in CAR expression between conditions with and without the use of a PhiC31 integrase system. However, the CAR expressions are above 50% at day 30 after transfection with the use of the PhiC31 integrase system. Contrarily, the CAR expression is hardly detected at day 30 in the control system. As the relative CAR mRNA expressions are compared, the PhiC31 integrase system achieved an approximately 10-fold enhancement of CAR expression in Jurkat cells. This suggested that the PhiC31 integrase system displayed a much higher efficiency at least in this specific case [65]. As a proof-of-concept, the introduction of nanobodies in CAR-T cells demonstrates similar activities to those of scFv from the CAR structure compatibility to cell functions.

Encouraged by the feasibility of anti-MUC1 nanobody in CAR constructs, nanobodies against TAG-72 [67] and HER2 [68] were constructed into second-/third-generation CARs subsequently. As many as 13 V_H_Hs have been identified to interact with the immobilized TAG-72 and TAG-72^+^ tumor cell, LS-174T. A V_H_H, N13, was selected to construct anti-TAG-72 CAR. The anti-TAG-72 CAR-T proliferates in specific response to the TAG-72-positive tumor cell lines of LS-174T and MCF7. In addition, the CAR-T cells show IL2 secretion and specific cell lysis upon tumor cell engagement. Five V_H_Hs were selected from an immunized camel with the use of HER2, which were joined into CD28-CD3ξ and CD28-OX40-CD3ξ signaling endodomains. Interestingly, the anti-HER2 CARs, constructed by the oligocolonal V_H_Hs or individual V_H_H, which are both transduced into Jurkat T cells. The oligoclonal V_H_H-CAR-engineered Jurkat T cells revealed a higher proliferation, IL2 secretion, and cytotoxicity.

The VEGFR2 (fetal liver kinase-1, FLK1, or kinase-insert domain receptor, KDR), belonging to the human VEGF receptor 1-3 family, is over-expressed on tumor vasculatures and is a promising anti-angiogenic target [81]. Although multiple chemical drugs and a Mab have been approved on marketing by targeting the VEGF/VEGR axis, challenges remain in their applications in T cell-based therapies. This is partially due to their immunosuppressive effects by inhibiting the activities of DC and effective T cells, enhancing the presence of Treg and MDSCs [82,83]. Nevertheless, a single dose of VEGFR2 CAR-engineered T cells significantly inhibited the growth of five different types of tumors in mice [84]. Therefore, if the numbers of adoptively transferred anti-VEGFR2 CAR-T cells are carefully escalated in the clinic, and safety mechanisms are introduced deliberately, the “On-target/Off-tumor” toxicity might be minimized [85].

A nanobody has been generated from two young male camels by injecting VEGFR2-overexpressing cells subcutaneously for six times at monthly interval. One nanobody, named 3VGR19, bound VEGFR2 with a *K_D_* value of 5.4 nM as measured by surface plasmon resonance (SPR). As demonstrated by FACS, this nanobody could selectively recognize VEGFR2-overexpressing tumor cells and primary endothelial cells (HUVECs). Following an in vitro assay, the capillary-like structures were successfully suppressed due to the blockage of VEGFR2 by the nanobody [86]. It indicated that 3VGR19 could block VEGFR2 signaling and thereby providing a potential application. The nanobody was further constructed into the second-generation CAR with the signaling domains of CD28 and CD3ζ. The resulting CAR-T cells display around 50% positive expression on the cell surface, which secretes IL-2 and IFN-γ and displays cytotoxic activity upon coculturing with VEGFR2-positive cells [69]. 

Prostate-specific membrane antigen (PSMA) is a classic target for prostate cancer that has attracted much attention for in drug discovery. A nanobody was generated from a llama immunized with four human-derived prostate cancer cell lines. The identified nanobody, JVZ-007, has the highest binding affinity with a *K_D_* value of ~27.4 nM [87]. JVZ-007 was constructed with the CD28 transmembrane, co-stimulatory domain and CD3ζ signaling domain, which responded to PSMA^+^ tumor cells, LNCaP and DU-145, by inducing IL2 and INF-γ secretion and increasing CD69 expression. It provided the support to CAR-T cells in PSMA-targeted immunotherapy [70].

The human V_H_ single-domain antibody library was firstly constructed by introducing both diverse human CDR2s and CDR3s plus synthetic CDR1s. The CDR1s are composed of random mutations of four putative solvent-accessible residues, A/D/S/Y. All these CDRs are combined into a human V_H_ single-domain framework, which results in a phage-display engineered library [88]. By this kind of library, a panel of V_H_ single domain antibodies with specificity to GPC2 were retrieved by the phage display and the lead nanobody displayed the binding affinity with a *K_D_* value of 9.8 nM. The produced CARs targeting GPC2 have been expressed in T cells isolated from eight individual healthy human donors. GPC2-specific CAR-T cells can efficiently lytic IMR5 neuroblastoma cells with high-level expression of GPC2. The CAR-T cells were generated from eight individual human donors to evaluate the killing ability. At an effector:target ratio of 8:1, GPC2-specific CAR-T cells reveal the cytotoxicty against IMR5 neuroblastoma cells ranged from 44% to 71%, with an average of 56%. In addition, the GPC2 CAR-T cells effectively suppressed the metastatic tumors or reduced the tumor size significantly in nude mice i.v. engrafted with IMR5 cells [71].

Two nanobodies, B3 and A12, were generated through immunizing alpaca by recombinant ectodomain of mouse PD-L1, which interact specifically with mouse PD-L1 on overlapping epitopes with the estimated affinities in the low nM range [89]. After adjoining A12 as PD-L1 binder in second generation CAR as shown in Table 1, the resulting CAR-T cells are capable of effectively lyse PD-L1 expressed cancer cell lines in a dose-dependent manner. These cell lines include B16 melanoma, an HPV16-transformed cell C3.43. and a colon adenocarcinoma MC38, thereby supporting its potential across a spectrum of cancers [72]. However, both macrophages and other immune cells express PD-L1 [90,91,92], which complicated the clinical application of this type of CAR-T therapy [72].

EIIIB is an alternatively spliced domain of fibronectin which is strongly expressed in tumors and during angiogenesis but with an extremely restricted distribution in normal adult tissues [93]. A nanobody library was generated from an alpaca immunized by a mixture of extracellular proteins (ECM), including full-length proteins, truncated domains, and peptides. After two rounds of panning, a nanobody, NJB2, was identified to bind specifically EIIIB with a *K_D_* value of 1.9 nM [94]. NJB2 was further utilized to generate CAR-T cells, displaying high transduction rate and specific cytotoxicity in vitro. The CAR-T cells were further proved to delay the tumor growth and improved the survival of WT C57BL/6 mice inoculated with B16 tumors. However, the treatment with CAR-T cells achieved minimal effects on the survival or tumor growth in the MC38 colon carcinoma model because of its low expression of EIIIB. In addition, low levels of immunogenicity against the CAR had been observed in a few mice, but no visible side effects developed upon repeated administration. No correlation had been observed between immunogenicity and animal survival [72]. 

Although CD38 expresses ubiquitously in many cells, especially in immune cells, it still remained an attractive target due to the extremely high expression levels in some malignant tumors [95]. The conventional CD38-specific mAb daratumumab [96] and isatuximab [97] have demonstrated their clinic efficacies in multiple myeloma. The advances have encouraged the development of CAR-T therapies. The purified recombinant C-terminal of CD38 was used to immunize llamas and the resulting nanobodies recognized CD38 at three different epitopes as revealed by crystallographic studies [98]. The nanobody against CD38, Nb-1G3, recognized the highly antigenic epitope located at the C-terminal region with a *K_D_* value of 4.11 nM [73]. The construction of Nb-1G3 with the CD8α hinge and transmembrane, 4-1BB co-stimulatory and CD3ζ activation domains to generate CAR-T cells, which demonstrated a high cytotoxic activity against CD38-positive fractions of T cells, B cells and natural killer cells in vitro. As expected, these CAR-T cells were able to effectively inhibit the tumor growth in NOD/SCID mice that were subcutaneously inoculated with RPMI 8226 cells [73]. In addition to CAR-T, another study utilized three nanobodies, WF211, MU1067 and JK36, targeting different epitopes, to construct CARs, and they showed similar dissociation rate constants ranging from 4.5 × 10^−3^ to 1.2 × 10^−4^s^−1^ [99]. After adjoining the nanobody to CARs separately, as shown in Table 1, they were transduced into the engineered human natural killer cell line by the CRISPR/Cas9, NK-92^CD38ko^, and stably expressed. The resulting CAR-NK cells provoked a specific, effective, and comparable cytotoxicity on CD38-expressing tumor cell lines independent of which nanobody was used or which epitope was targeted. Their cell lytic activities occurred in both, a time- and dose-dependent manner. These CD38-directed CAR-NK cells further displayed their activities against primary multiple myeloma cells from eight patients [74]. 

For the CD33 antigen, the soluble ectodomain was used to immunize a llama. The immunized PBMC served to construct a nanobody library for phage display selections. The achieved nanobody was constructed as listed in Table 1. It specifically lysed CD33-positive acute myeloid leukemia cell lines, including U937, HL60 and MOLM13 and Thp1. The xenograft model was established by intravenously injecting Thp1 cells in NGS mice for seven days. The engrafted mice were administrated by a single tail vein injection of anti-CD33 CAR-T cells. As compared with PBS, the treatments of CAR-T cells brought a reduction in tumor burden and improved survival rate [78]. As expected, anti-CD33 CAR-T cells caused an on-target/off-tumor effect because CD33 is also expressed on myeloid progenitors. 

CD20 is another attractive target. However, CD20 is a multiple-pass transmembrane protein and difficult to purify, besides its exposed extracellular parts are too short to form well-folded structural domains. Therefore, the researchers preferred to immunize a llama with an expression DNA vector encoding full-length CD20. Three nanobodies were identified from immunized Nanobody library, which all show vigorously cytotoxic activity on CD20^+^ cell lines, such as the Burkitt lymphoma cell line Raji and non-Hodgkin B lymphoblast cell line RL. In addition, the CAR-T cell targeting CD20 demonstrated significant in vivo function by eliminating the complete subcutaneous tumor in less than 20 days and prolonging the survival of mice remarkably [78].

The targets and corresponding CARs utilizing a nanobody listed in Table 1 supported the potential applications of such CARs by both cell-based assays and mouse models. However, their clinical effects are still unavailable except for BCMA. Two independent nanobody modules targeting BCMA have been developed and demonstrated their significant clinic effects on relapse/refractory multiple myeloma. One project has been developed by Pregene Biopharma and its CAR construct has been composed of a monovalent anti-BCMA nanobody, 4-1BB and CD3ζ (PRG1801) [77]. The other anti-BCMA CAR was constructed by Nanjing Legend Biotech, which employed two nanobodies recognizing BCMA in a bi-epitopic manner [75,76]. Both co-stimulatory and signaling domains are identical. Based on the public data, their clinical effects are both comparable to that of bb2121, which is developed by Bluebird with the use of an scFv to BCMA [100]. 

These nanobodies are generated from flexible immunization approaches, which ranges from expression DNA vector [78], recombinant proteins [99], stable cell lines [86] to cancerous tissues [101]. The V_H_H repertoires are established from peripheral blood lymphocytes and usually displayed on phage [86]. The panning processes are variable and depends on the availability of antigen. It is notable that the affinities of nanobodies are typically within a *K_D_* value of 1–10 nM for CAR construction. It provides the indication on the nanobody selection although the target expression and tissue distribution should be included in the consideration. However, the characterization of V_H_H are not generally included in the discovery of nanobodies for CAR-T therapy. It is postulated that antibody discovery follows the well-known procedure.

## 5. Nanobody in Advanced CAR

As shown in Figure 2, the aggregation of CARs due to the antigen binding domain, scFv, may induce excessive tonic signaling by an antigen-independent manner and eventually cause early exhaustion of T cells [54]. The nanobodies become an attractive and convenient module to develop the advanced and fourth-generation CARs. Table 2 shows the first nanobody-based CAR, targeting MUC1, that has been attempted on two intracellular domains. In addition, authors added a caspase8-induced suicide switch [102] to regulate the proliferation and to reduce potential unwanted side effects in vivo. During an in vitro assay, the suicide of CAR-T cells was triggered by adding a protein variant to a final concentration of 10 nM [66].

**Figure 3:**
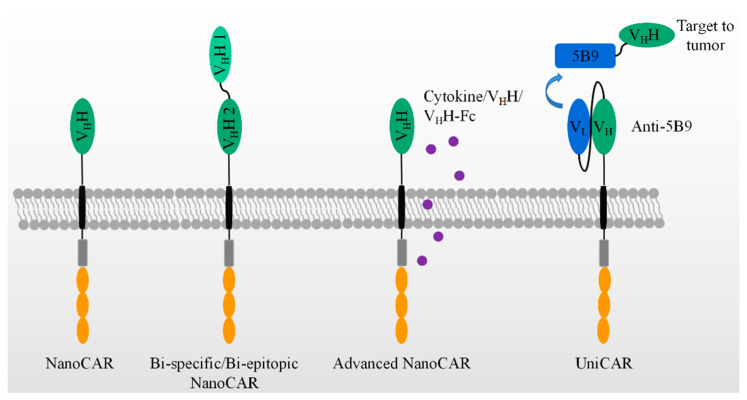
The application of V_H_H in advanced CAR-T therapies, which include bi-specific/epitopic, cytokine/V_H_H/V_H_H-Fc releasing, and UniCAR, etc.

As shown in Figure 3, bispecific CAR-T cells are believed to reduce the possibility of tumor escape by the loss of the target expression on tumor cells. V_H_Hs are devoid of light chains and are able to avoid domain swapping when multiple nanobodies are expressed simultaneously. A bispecific CAR-T cell was constructed by two nanobodies which target HER2 and CD20, respectively. Bispecific CAR-T cells display a similar activity to CD20- and HER2-expressing cells. They provide an option to circumvent tumor resistance after the loss of one antigen expression, although no clinical data are yet available [105]. In contrast, LCAR-B38M CAR-T cells revealed the feasibility of two nanobodies to target two epitopes on the extracellular domain of BCMA [99,100,106,107]. This construct could be manufactured and was effective in the clinic, as shown in Table 1. It demonstrated advantages and revealed the feasibility of replacing scFvs by nanobodies in generating bispecific CAR-T cells.

Besides fourth-generation CAR-T with the secretion of cytokines, many have been paid to explore the variable formats of antibody secretion with the use of NanoCAR-T therapies, as listed in Table 2. In comparison with scFv, the smaller size of a nanobody facilitates its insertion into a DNA construct. CAR-T cells were constructed with an anti-PD-L1 nanobody and an extra anti-CD47 V_H_H secretion system. As performed in PD-L1^+^ B16F10 cell, the INF-γ releasing and killing activity showed no difference between anti-PD-L1- and anti-47-secreting anti-PD-L1 CAR-T cells. However, as a C57BL/6 PD-L1 KO mouse was inoculated by B16F10 at day one, the treatments have been carried out by variable doses of anti-PD-L1 CAR-T cells at day 2, 6, and 12. The anti-CD47 secretion anti-PD-L1 CAR-T cells display better effects on the tumor sizes and mice survival than that of anti-PD-L1 CAR-T cells plus the systematic administration of anti-CD47 and anti-PD-L1 CAR-T-only groups. In addition, the anti-CD47 was secreted by CAR-T cells constructing an anti-EIIIB nanobody. The use of the treatments of these CAR-T cells on an in vivo model in C57BL/6 wild mouse showed an improved survival, as compared with that of anti-EIIIB CAR-T cells. Thus, anti-CD47-secreting anti-PD-L1 CAR-T cells improved the anti-tumor activity and achieved a significant survival benefit from an epitope-spreading mechanism. 

In Table 2, the authors further construct a series of V_H_H or/and V_H_H-Fc secreting anti-PD-L1/EIIIB CAR-T cells. No significant survival benefit has been observed in an in vivo mouse model. However, it is obvious that V_H_H or/and V_H_H- Fc can be locally delivered by the secretion of CAR-T cells [106].

Figure 3 also shows a universal modular platform termed UniCAR which was established to reduce the on-target/off-tumor side effects by the reversible and rapid control of CAR-T cell activity. The UniCAR technology splits the intracellular signaling and antigen-binding domain into two individual components. Structurally, the cellular part was composed of a scFv antibody, CD28α costimulatory, and CD3ζ signaling, as listed in Table 2. The scFv in the cellular part is able to specifically recognize a short peptide, 5B9. This construct was packaged into lentivirus and added to engineer human primary T cells. The target module included a tumor-specific nanobody and the short peptide, 5B9. This nanobody fused to the 5B9 tag was provided separately as a second and interchangeable component. In a mouse model, the UniCAR system is by itself totally inert in vivo unless the tumor-specific nanobody fused with the 5B9 tag is additionally administered [107]. Recently, an anti-EGFR nanobody fused with the 5B9 tag at its C-terminal was shown to activate and redirect the UniCAR-T cell to the EGFR-positive tumor cells both in vitro and in a mouse tumor xenograft model. By PET imaging, the nanobody can assemble to UniCAR reversibly [103]. Once the nanobody-5B9 was dissociated from UniCAR, the CAR-T cells were turned off, as expected. The same team further constructed a bivalent anti-EGFR nanobody that achieved a higher avidity than the monovalent nanobody. The bivalent nanobody fusion containing the 5B9 tag exerted its activity on the tumor cells, even those with a low antigen expression, after loading on UniCAR T cells. In comparison, the monovalent nanobody system could only redirect UniCAR T cells to tumor cells with a higher antigen expression [104].

## 6. The Clinical Effects of Different Anti-BCMA CAR-T Cells

Nanobodies have demonstrated their potential and feasibility in the discovery and development of CAR-T therapies. PRG1801 is composed of a monovalent anti-BCMA nanobody, 4-1BB, and CD3ζ [77,108], while the other anti-BCMA CAR-T developed by Nanjing Legend Biotech employed two nanobodies recognizing BCMA in a bi-epitopic manner [75,76,109,110]. Based on the published data, their clinical effects are both comparable to that of bb2121, which was developed by Bluebird with the use of an scFv targeted BCMA [100]. A comparison of the clinical data for three different BCMA-targeting CAR-T cells is listed in Table 3, including the CAR-T targeting BCMA using antigen recognition scFv, bi-epitopic tandem V_H_Hs, or single humanized V_H_H.

As shown in Table 3, the clinical dose of bb2121 was 150−300 × 10^6^ CAR-positive cells, the LCAR-B38M was 0.5−0.75 × 10^6^/kg body weight, and the PRG1801 was 2−10 × 10^6^/kg body weight. Based on the published data of bb2121, the Objective Response Rate (ORR) is 73%, and the Complete Response/stringent Complete Response (CR/sCR) is 31%. The data of LCAR-B38M from three independent clinical trials showed that their ORRs ranged from 80% to 94.8%. Their CRs/sCRs ranged from 56% to 76%. Another anti-BCMA nanobody-based CAR-T with a 2nd-generation CAR, PRG1801, has been used to treat 34 patients in a clinical study (NCT03661554). Within the median follow-up time of 12.5 months, 30 patients, representing 88.2%, had the best overall response, and 19 (55.9%) of them achieved complete remission [77,108].

The adverse events, including hematologic toxic effects, CRS, and neurotoxicity, for three anti-BCMA CAR-T therapies were controllable and comparable, although differences were found in the neurotoxicity. The CRS and neurotoxicity are the main adverse events related to CAR-T and were primarily caused by the excessive cytokine release [111,112,113]. BB2121 showed a mild severity and average frequency compared with other CAR-Ts, aside from the BCMA targeted, with an 84.4% overall CRS frequency and 5.4% > Grade 3 CRS and 17.8% overall neurotoxicity. LCAR-B38M showed more severe neurotoxicity, with a 20.6% overall occurrence and 10.3% Grade 3 or higher occurrence, which was probably due to the higher cytotoxic activity of the CAR-T with bi-epitopic tandem binding domains. PRG1801 revealed a relatively slight severity and lower frequency of adverse events, with 2.9% with Grade 3 CRS and no neurotoxicity observed. The safety data further showed promising results for nanobody-derived CAR-T in treating blood cancer, indicating that nanobodies with its special and beneficial characteristics can be broadly applied in CAR-T cellular drug development.

## 7. Summary

The anti-tumor effects of T cells have been recognized for about half a century. They evolved from “clinically effective/undefined targets” to “clinically ineffective/specific targets” to “clinically effective/defined targets”. CAR-T cell therapy is only one of its applications. Over a dozen targets have been modulated by nanobody-based CARs, which display similar activities to those of scFv. By the thoughtful application of its advantages, nanobody-based CARs have been extensively explored by broadening their therapeutic potential. Remarkably, no matter whether BCMA is modulated by a single nanobody or bivalent nanobodies, the constructed CAR-T cells displayed comparable clinical efficacies to those of scFv. In addition, LCAR-B38M, constructed with bivalent nanobodies, indicated the feasibility of developing and manufacturing bi-specific/valent CARs. 

## Figures and Tables

**Table 1:** The applications of nanobodies in CAR-T therapies.

Target	CAR Structure				Reference
	Spacer	Transmembrane	Costimulatory	Signaling	
MUC1	IgG3-Fc&Hinge	CD28	CD28	CD3ζ	[64,65]
	IgG3-Fc&Hinge-Hinge				
	FCγRII Hinge				
	IgG3-Fc&Hinge	CD28	CD28-OX40	CD3ζ	[66]
	IgG3-Fc&Hinge-Hinge				
TAG-72	IgG3-Fc&Hinge	CD28	CD28	CD3ζ	[67]
	IgG3-Fc&Hinge-Hinge				
	IgG3-Fc&Hinge		CD28-OX40	CD3ζ	
	IgG3-Fc&Hinge-Hinge				
HER2	IgG3-Fc&Hinge	CD28	CD28	CD3ζ	[68]
	IgG3-Fc&Hinge-Hinge				
	IgG3-Fc&Hinge		CD28-OX40	CD3ζ	
	IgG3-Fc&Hinge-Hinge				
VEGFR2	IgG1-Fc	CD28	CD28	CD3ζ	[69]
PSMA	IgG1-Fc	CD28	CD28	CD3ζ	[70]
GPC2 ^a^	CD8α	CD8α	4-1BB	CD3ζ	[71]
PD-L1	CD8α	CD8α	CD28	CD3ζ	[72]
EIIIB					
CD38	CD8α	CD8α	4-1BB	CD3ζ	[73]
CD38 ^b^	IgG4-Hinge	CD28	CD28-4-1BB	CD3ζ	[74]
BCMA ^c^	CD8α	CD8α	4-1BB	CD3ζ	[75,76]
BCMA	CD8α	CD8α	4-1BB	CD3ζ	[77]
CD20	CD8α	CD8α	4-1BB	CD3ζ	[78]
CD33	CD8α	CD8α	4-1BB	CD3ζ	

^a^ Human VH single-domain antibody; ^b^ CAR-NK; ^c^ bi-epitopic tandem V_H_H.

**Table 2:** The applications of nanobodies in advanced CAR-T therapies.

Target	CAR Structure					Reference
	Spacer	Transmembrane	Costimulatory	Signaling	New Function	
MUC1	IgG3-Fc&Hinge-Hinge	CD28	CD28-OX40	CD3ζ	iCaspase 8	[66]
EGFR	Anti-E5B9-CD28	CD28	CD28	CD3ζ	UniCAR	[103]
	Anti-E5B9-CD28	CD28	CD28	CD3ζ	UNiCAR & Bivalent V_H_H	[104]
CD20&HER2	IgG1-Fc	CD28	CD28	CD3ζ	Bispecific	[105]
PD-L1	CD8α	CD8α	CD28	CD3ζ	secrete anti-CD47	[106]
	CD8α	CD8α	CD28	CD3ζ	secrete anti-CD47-Fc	
	CD8α	CD8α	CD28	CD3ζ	secrete anti-CTLA4-Fc& anti-CD47	
EIIIB	CD8α	CD8α	CD28	CD3ζ	secrete anti-PD-L1	
	CD8α	CD8α	CD28	CD3ζ	secrete anti-CD47	
	CD8α	CD8α	CD28	CD3ζ	secrete anti-CTLA4-Fc	

**Table 3:** The summary of clinical efficacies and side effects from three independent anti-BCMA CAR T cells.

	bb2121 (128/33 Cases) [100]	LCAR-B38M (57 Cases) [75]	LCAR-B38M (17 Cases) [76]	LCAR-B38M/JNJ-4528 (97 Cases) [110]	PRG1801 (34 Cases) [77,108]
**Antigen Module**	Humanized scFv	Bi-epitopic tandem V_H_Hs			Single humanized V_H_H
**Dosage**	50 × 10^6^, 150 × 10^6^, 450 × 10^6^, 800 × 10^6^	Median dose, 0.5 × 10^6^ cells/kg	Median dose, 0.7 × 10^6^ cells/kg	Median dose, 0.75 × 10^6^ cells/kg	Median dose, 5 × 10^6^ cells/kg
**Efficacy**	ORR, 73%; CR/sCR, 31%	ORR, 88.0%; CR, 68.0%	ORR, 88.2%; CR, 76.5%	ORR, 94.8%; sCR, 56.0%	ORR, 88.2%; sCR, 55.9%
**Grade 3 or higher hematologic toxic effects**	Neutropenia, 85%; (33 cases)	Leukopenia, 30%	Neutropenia, 47.1%	Neutropenia, 90.7%	Neutropenia, 44.1%;
	Leukopenia, 58%; (33 cases)	Thrombocytopenia, 23%	Leukopenia, 58.8%	-	Leukopenia, 32.4%
	Anemia, 45%; (33 cases)	-	Thrombocytopenia, 17.6%	Anemia, 68%	Anemia, 20.6%
	Thrombocytopenia, 45% (33 cases)	-	-	Thrombocytopenia, 59.8%	Thrombocytopenia, 38.2%
**Neurotoxicity**	Grade 1 or 2, 14.8%;	Grade 1, 1.8%	-	Grade 1 or 2, 10.3%	-
	Grade 4, 3%			Grade 3 or higher, 10.3%	
**CRS**	Grade 1 or 2, 79%;	Grade 1 or 2, 83%;	Grade 1 or 2, 58.8%	Grade 3 or higher, 4.1%	Grade 1 or 2, 82.4%
	Grade 3, 5.4%	Grade 3, 7%	Grade 3 or higher, 41.2%		Grade 3, 2.9%

## Data Availability

Patient-related data not included in the paper were generated as part of a clinical trial and may be subject to patient confidentiality. The datasets used and/or analyzed during the current study are available from the corresponding author on reasonable request.
